# Regional asymmetry in financial markets: Pricing of skewness risk in the Thai stock market

**DOI:** 10.1371/journal.pone.0336697

**Published:** 2025-11-12

**Authors:** Tran Trong Huynh, Bui Thanh Khoa

**Affiliations:** 1 FPT University, Ha Noi, Vietnam; 2 Industrial University of Ho Chi Minh City, Ho Chi Minh City, Vietnam; Chosun University, KOREA, REPUBLIC OF

## Abstract

This study examines the role of skewness as a priced risk factor in the Thai stock market, drawing on both rational and behavioral asset pricing theories. Theoretically, skewness captures return asymmetry that investors may price due to downside risk aversion or misprice due to lottery-seeking behavior. Using daily data from 2005 to 2024, we construct decile portfolios based on lagged skewness and find a significant negative relationship between skewness and future returns. A long-short strategy that buys stocks with the lowest skewness and shorts those with the highest yields an average excess return of 1.017% monthly. Standard models (including CAPM, Fama-French three-factor, Carhart four-factor, and Fama-French five-factor frameworks) fail to explain this anomaly, as the alpha of the skewness-sorted portfolio remains significantly positive in all specifications. Fama-MacBeth regressions further confirm skewness as a priced risk factor in the cross-section of returns. These findings challenge the semi-strong form of the Efficient Market Hypothesis (EMH) and highlight persistent market inefficiencies in Thailand, where short-selling constraints, behavioral biases, and asymmetric return expectations are prevalent. This study contributes to the emerging market asset pricing literature and offers practical insights for portfolio construction and regulatory design.

## 1. Introduction

Asset pricing models are fundamental tools for understanding the determinants of stock return. Foundational frameworks such as the Capital Asset Pricing Model [1], Fama-French three-factor model [2], Carhart’s four-factor model [3], and Fama-French five-factor model [4] assume that investors are primarily concerned with the first two moments of return distributions— the mean and variance. These models posit that expected returns are driven by systematic risk factors, such as market risk, firm size, value characteristics, profitability, and investment intensity.

However, a growing body of literature suggests that higher-order moments, particularly skewness (the asymmetry of return distributions), play an important role in shaping investor preferences and asset prices. Skewness captures the probability of extreme outcomes: negative skewness indicates a greater chance of large losses, whereas positive skewness implies a higher likelihood of large gains. Investors generally exhibit asymmetric risk preferences; they demand compensation for bearing downside risk (negative skewness) but may overvalue assets with lottery-like payoffs (positive skewness) [5]. This behavior introduces a potential skewness premium in financial markets, whereby assets with less favorable skewness profiles must offer higher expected returns as compensation [6].

Empirical evidence on whether skewness is priced remains mixed and is context-dependent. In developed markets, several studies [7,8] find that positively skewed stocks tend to underperform, suggesting that investors overpay for the upside potential. In contrast, emerging markets appear to be more sensitive to skewness, with stronger pricing effects observed. Research in less efficient markets, such as South Africa and Thailand, has shown that behavioral biases, limited arbitrage, and market frictions may amplify the role of return asymmetry in asset pricing [9,10].

Despite these findings, traditional asset-pricing models do not formally incorporate skewness as a systematic risk factor. The CAPM ignores higher moments entirely, whereas the Fama-French and Carhart extensions focus on linear risk exposures. Consequently, pricing anomalies related to skewness may persist, particularly in environments where return distributions deviate significantly from normality and investors respond to tail risk. This study aims to examine whether skewness risk is a priced factor in the Thai stock market, an emerging economy characterized by relatively high volatility, speculative trading behavior, and evolving market structures. Thailand offers a unique testing ground for evaluating the role of skewness because of the presence of both institutional inefficiencies and growing investor participation. Specifically, we investigate whether return asymmetry influences stock pricing in a manner consistent with the theoretical predictions of skewness premiums.

To empirically assess this, we construct portfolios sorted by lagged skewness and analyze their return patterns using standard asset pricing models. Additionally, we adopt Fama and MacBeth’s [11] two-step regression methodology to examine whether skewness systematically explains the cross-section of stock returns after accounting for market beta. By focusing on a market that is neither fully developed nor severely segmented, our analysis contributes to a broader understanding of skewness in transitional capital markets. This research is grounded in the theoretical framework developed by Siddique and Harvey [12], who extended the CAPM to account for conditional skewness, and is further supported by recent advancements in the pricing of moment risk premia [13,14]. We hypothesize that if investors require compensation for skewness exposure, skewness should manifest as a priced component in expected returns, particularly in markets where traditional linear models fall short.

The remainder of this paper is organized as follows. Section 2 reviews the theoretical foundations and empirical findings of skewness in asset pricing. Section 3 describes the data and methodology, including portfolio construction, regression analysis, and model selection. Section 4 presents the empirical results, examining the performance of skewness-sorted portfolios and the robustness of the findings. Section 5 discusses the implications of the results, and future research.

## 2. Literature review

Skewness, a measure of asymmetry in return distributions, has been increasingly recognized as a critical risk factor in asset pricing [14]. Traditional asset pricing models, such as the Capital Asset Pricing Model and the Fama-French three-factor and five-factor models, assume that investors are primarily concerned with the mean and variance. However, subsequent studies have highlighted the importance of higher moments, particularly skewness, in determining expected returns [15]. Theoretically, risk-averse investors demand compensation for holding assets with negative skewness because such assets expose them to large potential downside risks. This risk compensation framework suggests that systematic negative skewness should be priced into the expected returns, leading to a skewness premium.

Siddique and Harvey [12] extended the CAPM framework by incorporating conditional skewness, demonstrating that negatively skewed assets require higher expected returns as compensation for downside risk. Similarly, Vendrame, Guermat [13] argue that investors exhibit preferences for higher-order moments, suggesting that systematic skewness is an integral component of asset pricing models. Further theoretical advancements incorporate the concept of skewness risk premium, which is derived from the difference between physical and risk-neutral skewness [14]. This framework suggests that investors in equity and options markets require compensation for bearing skewness risk, particularly during periods of high-risk aversion. The presence of a significant skewness risk premium in asset pricing models implies that skewness is not merely an artifact of return distributions but a distinct and economically meaningful risk factor. Beyond traditional asset pricing models, a growing body of literature has explored the role of risk-neutral skewness in security pricing. Risk-neutral skewness has long been linked to volatility smirks observed in options markets [16]. Studies such as Conrad, Dittmar [7], Chang, Christoffersen [17] use option-implied skewness data to demonstrate a significant negative relationship between risk-neutral skewness and subsequent stock returns. These findings suggest that skewness risk is priced in both equity and derivative markets.

The concept of a skewness risk premium has also gained traction, where skewness differences between realized and risk-neutral measures create arbitrage opportunities [18]. More recent empirical work by Elyasiani and Gambarelli [9] examines the moment risk premium, and demonstrates that the skew risk premium is negatively related to stock returns, further reinforcing the idea that skewness is a crucial factor in asset pricing. Additionally, behavioral finance research supports the idea that variance and skewness influence investor decision-making different ways. Symmonds, and Wright [19] used fMRI brain scans to show that individuals process variance and skewness as separate risk components, with variance linked to the parietal cortex and skewness associated with the prefrontal cortex and ventral striatum. This neuro-scientific perspective aligns with asset pricing theories that consider skewness as a separate risk factor influencing investor behavior and market returns.

Empirical studies provide mixed evidence on skewness risk pricing across different markets. Research in developed markets suggests that positively skewed stocks tend to be overvalued because of investors’ lottery-like preferences. Boyer, and Mitton [8] document that stocks with higher expected idiosyncratic skewness exhibit lower subsequent returns, indicating that investors are willing to accept lower returns in exchange for the possibility of extremely positive payoffs. Langlois [20] showed that systematic and idiosyncratic skewness play distinct roles in explaining expected stock returns. By employing a novel methodology that utilizes a large set of predictors, this study forecasts the cross-sectional ranks of systematic and idiosyncratic skewness, demonstrating that these ranks are more predictable than their actual values. Compared to other ex-ante measures of systematic skewness, these forecasts create a significant spread in the ex-post systematic skewness. The findings reveal that the predicted systematic skewness risk factor carries a substantial and robust risk premium, ranging from 6% to 12% annually. In contrast, the impact of idiosyncratic skewness on stock pricing is less consistent, suggesting that systematic skewness is a more critical factor in asset pricing than idiosyncratic skewness. In contrast, the emerging markets exhibit different patterns. Studies on markets such as Vietnam, Thailand, and South Africa suggest that skewness risk plays a more prominent role due to market inefficiencies and higher risk aversion. Steyn and Theart [10] show that in the Johannesburg Stock Exchange, stocks with positive skewness achieve higher risk-adjusted returns, contrary to the findings in developed markets. Drerup, Wibral [21] provide empirical evidence that investors tend to allocate more of their portfolios to assets that they expect to have higher skewness. This finding highlights the crucial role of skewness in investment decisions, as investors are willing to take on greater risks in pursuit of extreme returns, even when controlling for other factors such as expected returns and variance.

In the Thailand stock market, Cheuathonghua, Wattanatorn [22] highlighted the significant impact of stock liquidity on tail risk, demonstrating that higher liquidity is associated with greater downside risk. Their findings suggest that increased liquidity facilitates speculative trading, leading to more extreme price fluctuations and amplifying the risk of stock price crashes. This aligns with broader research on emerging markets, where liquidity-driven volatility exacerbates the return asymmetry. By employing multiple liquidity measures and addressing endogenetic concerns through a two-stage least squares regression, their study provides robust evidence that stock liquidity plays a crucial role in shaping tail-risk dynamics in the Thai market. Similarly, Wattanatorn and Padungsaksawasdi [23] emphasize the importance of systematic skewness in determining stock price crash risk. Their findings reveal a negative relationship between co-skewness and crash risk, indicating that stocks with higher co-skewness are less likely to experience extreme downward-price movements. This study reinforces this relationship by accounting for factors such as stock liquidity, earnings management, and financial opacity. Using a two-stage least squares methodology to address endogenetic, their study provides strong empirical support for the role of systematic skewness in influencing tail risk in emerging markets. These findings further highlight the necessity of incorporating skewness as an independent risk factor in asset pricing frameworks, particularly in markets where traditional models fail to capture skewness-based return variations.

Although there has been extensive research on skewness, studies examining the role of skewness in asset pricing remain limited in Thailand. This study aims to fill this research gap by investigating whether skewness acts as a significant pricing factor and whether a skewness-related anomaly exists in the Thai stock market. By incorporating skewness into asset pricing models, this study contributes to a broader understanding of how return asymmetry influences expected returns in emerging markets. To address these questions, this study formulates three key hypotheses regarding the role of skewness in asset pricing:

- **H1: The long-short skewness portfolio generates significant excess returns.** This hypothesis posits that a long-short portfolio, constructed by taking a long position in low-skewness stocks and a short position in high-skewness stocks, should yield positive and statistically significant returns. If investors demand compensation for bearing negative skewness risk, portfolios that exploit this pricing inefficiency will exhibit abnormal returns.
- **H2: The existence of a skewness-related anomaly in asset pricing.** If skewness risk is systematically mispriced, traditional asset pricing models (CAPM, FF3, FF4, and FF5) should fail to fully explain the returns of the long- and short-term skewness portfolios. A statistically significant alpha (intercept) in multifactor regressions indicates the presence of an anomaly, suggesting that skewness is a missing factor in conventional asset pricing frameworks.
- **H3: Skewness is a significant determinant of the expected stock returns.** This hypothesis is tested using Fama-MacBeth regressions, where the cross-sectional pricing of skewness is evaluated after controlling for market risk. If the coefficient of skewness is statistically significant, it provides robust evidence that skewness is a priced risk factor in emerging markets.

The findings of this study contribute to the growing body of literature on asset pricing anomalies by providing empirical evidence that skewness plays a fundamental role in determining stock returns, particularly in Thailand, where traditional risk factors may not fully capture return variations.

## 3. Methodology

### 3.1. Data and variable description

This study utilizes daily adjusted closing prices of stocks listed on the Stock Exchange of Thailand (SET) over the period 2005–2024. The data were sourced from Yahoo Finance and included all stocks listed for at least two years. The data for this study were sourced from Yahoo Finance because of its accessibility, historical coverage, and widespread use in the academic literature. While institutional databases such as Bloomberg or Refinitiv may offer greater precision, access to such sources is limited. To validate data reliability, we cross-checked all stocks with alternative public sources such as Investing.com and SET’s official portal, confirming consistent price and return information. Nonetheless, we acknowledge that Yahoo Finance may omit certain corporate actions or adjustments, which constitutes a limitation of this study. Companies with a listing history of less than 24 months are excluded to ensure sufficient data for skewness estimation and return calculations. Daily stock return for stock i on day j of month t is computed as:


$$r_{i,j,t}=log\left(\frac{P_{i,j,t}}{P_{i,j-\text{1},t}}\right)\times \text{1}\text{0}\text{0}$$ (1)

where P_i,j,t_ represents the adjusted closing price of stock ii on day j in month t.

- Monthly stock return for stock ii at the end of month t is calculated as:

$$ret_{i,t}=log\left(\frac{P_{i,t}}{P_{i,t-\text{1}}}\right)\times \text{1}\text{0}\text{0}$$ (2)

where P_i,t_ is the adjusted closing price at the end of month t.

- The skewness of stock i in month t is computed using daily returns within the month:

$$\text{ske}{\text{w}}_{i,t}=\frac{\text{1}}{n_{j}}\sum_{j=\text{1}}^{n_{j}}r_{i,j,t}^{\text{3}}$$ (3)

where r_i,j,t_ is the daily return of stock i on day j, and n_j_ is the number of trading days in month t.

This measure captures the third moment of the return distribution, reflecting the degree of asymmetry in the stock returns.

- Risk-Free Rate and Market Factors

The risk-free rate is represented by the 1-year government bond yield. The market excess return (Mkt) is calculated as


$$Mkt_{t}=\text{Return}\;\text{of}\;\text{the}\;\text{STI}\;\text{Inde}{\text{x}}_{t}-R_{f}$$ (4)

The SMB (Small Minus Big) and HML (High Minus Low) factors are constructed following Fama and French’s [4] methodology. The momentum (MOM) factor captures the return difference between winners and losers [3]. The CMA (Conservative Minus Aggressive) and RMW (Robust Minus Weak) factors are also computed according to the Fama-French five-factor model.

### 3.2. Portfolio construction and anomalies analysis

To investigate whether skewness risk is priced in the Thai stock market, this study constructs decile portfolios based on the previous month’s skewness. At the end of each month, all stocks are ranked in ascending order based on their lagged skewness. The stocks are then divided into ten equal-sized deciles, labeled from P1 to P10. The first decile (P1) consists of stocks with the lowest skewness (most negatively skewed), while the last decile (P10) contains stocks with the highest skewness (most positively skewed). A long-short portfolio (P11) is constructed to capture the return spread between low- and high-skewness stocks. This portfolio takes a long position in P1 and a short position in P10, thus isolating the effect of skewness on returns. The return of the long-short portfolio is computed as the equal-weighted return difference between the two deciles. Specifically, at the beginning of each month, an investor buys all stocks in P1 and simultaneously sells all stocks in P10, assuming an equal capital allocation across all stocks within each decile.

Although short selling is legally permitted in Thailand under certain conditions, it remains restricted and subject to high transaction costs. For this study, we assume a frictionless market in which short selling is unrestricted and transaction costs are negligible. This assumption allows for a theoretical assessment of the skewness risk premium, although real-world implementation may be subject to practical constraints. To evaluate the statistical significance of the long- and short-term portfolio performance (Hypothesis H1), we conduct a t-test on the mean returns of P11. The test examines whether the portfolio’s average return is significantly different from zero. A statistically significant result would indicate that skewness-based portfolio strategies generate abnormal returns, suggesting that skewness is a priced risk factor in the Thai stock market.

To further investigate whether the long-short skewness portfolio (P11) exhibits abnormal returns, this study employs multifactor regressions to assess whether traditional asset-pricing models can explain the skewness-based return premium. The CAPM, Fama-French three-factor (FF3), Carhart four-factor (FF4), and Fama-French five-factor (FF5) models are used to analyze the return predictability of P11 and evaluate the existence of an anomaly related to skewness. Regressions are conducted to determine whether skewness is an independent risk factor that contributes to expected returns beyond conventional market risk factors. The regression models take the following form:


$$(CAPM):E\left(P_{\text{1}\text{1}}\right)=\alpha_{CAPM}+\beta_{Mkt}Mkt$$ (5)

$$(FF\text{3}):E\left(P_{\text{1}\text{1}}\right)=\alpha_{FF\text{3}}+\beta_{Mkt}Mkt+\beta_{SMB}SMB+\beta_{HML}HML$$ (6)

$$(FF\text{4}):E\left(P_{\text{1}\text{1}}\right)=\alpha_{FF\text{4}}+\beta_{Mkt}Mkt+\beta_{SMB}SMB+\beta_{HML}HML+\beta_{MOM}MOM$$ (7)

$$(FF\text{5}):E\left(P_{\text{1}\text{1}}\right)=\alpha_{FF\text{5}}+\beta_{Mkt}Mkt+\beta_{SMB}SMB+\beta_{HML}HML+\beta_{CMA}CMA+\beta_{CMA}RMW$$ (8)

Hypothesis H2 states that skewness is a significant determinant of expected stock return. The hypothesis is supported if alpha (α) remains statistically significant across all asset pricing models.

### 3.3. Fama-MacBeth regression

To test H3, this study employs the Fama-MacBeth’s two-step regression approach, which is widely used in empirical asset pricing to examine whether a particular factor systematically influences expected stock returns. The methodology is implemented as follows: In the first stage, monthly cross-sectional regressions are performed, where the dependent variable is the expected stock return (ret_𝑖,𝑡+1_), and the independent variables include the estimated market beta and the skewness of individual stocks (skew_i,t_):


$$ret_{i,t+\text{1}}=\gamma_{\text{0}}+\gamma_{skew}s\text{ke}{\text{w}}_{i,t}+\gamma_{Mkt}\beta_{\text{Mkt},i}+\varepsilon_{i,t}$$ (9)

where the stock’s market beta (β_Mkt_) was estimated by running time-series regressions based on the CAPM model using a 24-month rolling window, and ε_i,t_ are error terms.

The second step involves calculating the time-series average of the estimated coefficients (γ_skew_ and γ_Mkt_) from the first-stage regressions. A statistically significant and negative coefficient on skewness (γ_skew_) provides strong evidence that stocks with higher skewness tend to have lower expected returns, confirming that skewness is a priced risk factor in the Thai stock market. This result supports the risk compensation hypothesis, suggesting that investors require a return premium to hold stocks with greater downside risk.

## 4. Results and discussion

### 4.1. The descriptive statistics and correlation matrix

The period from 2005 to 2024 marks significant economic and financial development in Thailand. The Stock Exchange of Thailand (SET) has experienced multiple market cycles, including the aftermath of the Asian Financial Crisis, the Global Financial Crisis (2008−2009), political instability, and the impact of the COVID-19 pandemic. Despite these challenges, the Thai equity market has shown resilience, supported by robust domestic consumption, industrial growth, and increasing foreign investment. The financial sector, in particular, has played a crucial role in shaping market trends, whereas government policies have influenced investment dynamics, capital flows, and overall stock market performance. Given these conditions, understanding skewness risk in asset pricing is highly relevant, as market participants often face extreme downside risks and asymmetric return distributions in emerging markets such as Thailand.

Table 1 presents summary statistics of the key risk factors and return variables. The market factor (Mkt) exhibits an average monthly return of 0.257%, with a high standard deviation of 6.409%, indicating substantial market volatility during the period. The size factor (SMB) has a slightly negative mean (−0.019%), suggesting that, on average, smaller firms underperformed relative to the larger firms. Similarly, the momentum factor (MOM) shows a positive mean of 0.556%, implying that past winners tend to continue their performance, which aligns with the momentum effects observed in the emerging markets. The skewness variable (skew) exhibits extreme variation, with an average of 17.641, and a high standard deviation of 616.223, suggesting that stock returns in Thailand exhibit substantial asymmetry. The skewness distribution is heavily right-skewed (21.875), implying that a small subset of stocks experienced large positive skewness, consistent with lottery-like stock behavior. The return variable (ret) is also positively skewed (2.787) but highly volatile, with a standard deviation of 12.770%, highlighting the high-risk nature of the Thai stock market. The minimum and maximum values for skewness range from −50,163.956 to 46,185.912, reflecting the presence of stocks with extreme-return distributions. This substantial dispersion underscores the importance of considering skewness in asset pricing models, particularly in markets where speculative trading and high-volatility events occur frequently.

**Table 1:** The summary statistics of the variables.

	*Mkt*	*SMB*	*HML*	*MOM*	*RMW*	*CMA*	*ret*	*skew*
Mean	0.25	−0.13	0.268	0.627	0.086	0.302	0.166	9.914
Median	0.593	−0.075	0.007	0.508	0.142	0.248	0	0.073
St. Dev.	5.998	3.406	2.023	3.936	2.155	2.714	11.89	421.978
Skewness	−0.861	−2.232	0.708	−0.893	−0.327	0.556	1.088	−6.311
Minimum	−36.181	−29.365	−4.777	−24.751	−7.593	−10.867	−223.253	−50163.96
Maximum	21.022	7.957	11.735	17.19	6.769	13.119	287.44	41224.86

Table 2 presents the correlation matrix of the key risk factors and the long-short skewness portfolio P11. The market factor (Mkt) is weakly negatively correlated with P11 (−0.047), indicating that skewness-based portfolio returns are not systematically driven by market movements. This suggests that the long-short skewness strategy captures an independent return component, separate from the overall market risk. The size factor (SMB) exhibits a small positive correlation (0.026) with P11, suggesting that smaller firms have slightly higher skewness-based return differentials. This could be attributed to the fact that small-cap stocks often exhibit greater return asymmetry and speculation. The value factor (HML) has a weak negative correlation (−0.033) with P11, implying that value stocks tend to have lower skewness-based return spreads than growth stocks. This aligns with the idea that growth stocks, which are often highly speculative, may exhibit higher skewness. The momentum factor (MOM) has the highest positive correlation with P11 (0.080). This suggests that stocks with strong past performances tend to exhibit greater skewness-based return spreads, possibly due to investor overreaction to past returns. This aligns with the findings of behavioral finance, where momentum effects can contribute to asymmetric return distributions. The profitability (RMW) and investment (CMA) factors show weak negative correlations (−0.003 and −0.058, respectively) with P11. This suggests that neither firm profitability nor investment policies strongly influences the skewness premium in Thailand.

**Table 2:** The correlation between factors.

	*P11*	*Mkt*	*SMB*	*HML*	*MOM*	*CMA*	*RMW*
P11	1						
Mkt	−0.007	1					
SMB	0.032	−0.194	1				
HML	0.049	−0.076	0.030	1			
MOM	−0.138	−0.231	−0.094	−0.058	1		
CMA	0.032	−0.189	0.132	−0.012	0.002	1	
RMW	−0.092	−0.284	−0.488	−0.038	0.395	−0.293	1

### 4.2. Portfolio analysis

All stocks listed on the Stock Exchange of Thailand (SET) from 2005 to 2024 are sorted at the end of each month based on their lagged skewness, calculated from daily returns over the previous month. At the end of month *t*, the skewness of each stock is measured using its daily return distribution in month *t* − 1. The stocks are then ranked in ascending order and divided into ten decile portfolios (P1 to P10), where P1 contains stocks with the lowest skewness (most negatively skewed), and P10 consists of stocks with the highest skewness (most positively skewed). Each portfolio is rebalanced monthly to ensure that the ranking remains reflective of the most recent skewness estimates.

To analyze the pricing of skewness risk, an equal-weighted long-short portfolio (P11) is constructed by going long in P1 and short in P10, thereby capturing the return differential between stocks with low and high skewness. This strategy isolates the impact of skewness on asset pricing and allows for an assessment of whether investors demand compensation for exposure to negative skewness risk.

To examine whether skewness is a priced risk factor in asset pricing, this study analyzes the performance of decile portfolios sorted by skewness exposure. Table 3 presents the descriptive statistics for the ten decile portfolios (P1 to P10) and the long-short portfolio (P11), which captures the return differential between stocks with the lowest and highest skewness. The results indicate a clear pattern: stocks in the lowest skewness decile (P1) tend to earn higher returns than those in the highest skewness decile (P10) do. Specifically, P1 yields an average return of 0.127% per month, whereas P10 experiences a negative average return of −0.890% per month.

**Table 3:** Summary statistics of excess returns across portfolios.

Portfolio	Obs.	Mean	St. Err.	Min	Max	t-value	p-value
P1	240	0.127	0.425	−28.862	28.588	0.298	0.766
P2	240	−0.130	0.433	−41.127	33.520	−0.301	0.764
P3	240	−0.162	0.379	−36.443	27.125	−0.428	0.669
P4	240	0.259	0.366	−41.255	27.886	0.708	0.480
P5	240	0.420	0.337	−36.257	19.368	1.244	0.215
P6	240	0.285	0.324	−27.928	22.059	0.881	0.379
P7	240	0.586	0.307	−18.573	14.482	1.906	0.058
P8	240	0.492	0.320	−21.651	15.200	1.536	0.126
P9	240	0.273	0.397	−36.977	16.849	0.688	0.492
P10	240	−0.890	0.419	−37.032	19.927	−2.124	0.035
P11	240	1.017	0.258	−9.967	22.023	3.942	0.000

The return differential between these two deciles, represented by the long-short portfolio (P11), is statistically significant, with an average monthly return of 1.017% and a t-statistic of 3.942 (p-value = 0.000). The significance of P11 suggests that investors who take a long position in low-skewness stocks and short high-skewness stocks earn positive abnormal returns. This finding is consistent with the risk-based interpretation of skewness pricing, where stocks with high negative skewness require a return premium to compensate for exposure to downside risk. To formally test whether the long-short portfolio (P11) delivers a significantly positive return.

The t-value of 3.942 and p-value of 0.000, reported in Table 3, indicate that we can reject the null hypothesis at the 1% significance level, providing strong statistical evidence that skewness is a priced factor in the Thai stock market. These results support the notion that skewness risk is an essential component of asset prices. The negative relationship between skewness and expected returns suggests that investors demand compensation for bearing downside risk, aligning with the theoretical predictions of Boyer, Mitton [8]. However, this finding contrasts with Steyn and Theart [10], who document a positive relationship between skewness and stock returns on the Johannesburg Stock Exchange. Their study suggests that in emerging markets with high market inefficiencies, investors may prefer positively skewed assets due to their lottery-like payoffs, leading to higher expected returns for stocks with greater skewness. In contrast, our findings indicate that in the Thai stock market, investors perceive negatively skewed stocks as riskier and require a return premium to hold them, reinforcing the traditional risk compensation hypothesis. This discrepancy highlights the potential influence of market structure, investor sentiment, and behavioral biases on skewness risk pricing across different emerging markets.

### 4.3. Multifactor model regression and Fama-Macbeth regression analysis

The multifactor regression analysis evaluates whether the long-short skewness portfolio generates abnormal returns that standard asset pricing models cannot explain. By regressing P11 on the CAPM, Fama-French 3-Factor, Carhart 4-Factor (including momentum), and Fama-French 5-Factor models, we assess the existence of an anomaly in asset pricing. To ensure robust statistical inference, the Newey-West standard errors are applied to correct for heteroskedasticity and autocorrelation [24].

The regression results, reported in Table 4, indicate that the alpha (α) coefficients across all models remain positive and statistically significant at the 1% level. Specifically, the CAPM model’s estimated alpha is 1.0123 with a t-value of 3.978, suggesting that P11 generates a positive abnormal return beyond what the market factor (Mkt) explains. This result is consistent across all multifactor models, with alpha ranging from 1.0074 (FF3) to 1.0985 (FF5), and all estimates remain statistically significant at the 1% level or better. The persistence of a significant alpha across models provides strong evidence of an anomaly, implying that traditional risk factors do not capture skewness. This result implies that H2 is supported. When comparing the model performance, the R² values suggest that none of the models explain a substantial proportion of P11’s return variation. The CAPM model has the lowest explanatory power with an *R*^2^ of 0.0023, meaning that market risk alone provides almost no explanation for the skewness-based return premium. The FF3 model slightly improves fit with an *R*^2^ of 0.0029, indicating that size (SMB) and value (HML) factors contribute negligibly to explaining P11. The Carhart 4-Factor model, which includes the momentum factor (MOM), increases R^2^ to 0.0138, suggesting that momentum is marginal in explaining skewness-based return. However, the FF5 model, which incorporates profitability (RMW) and investment (CMA) factors, yields the highest R^2^ (0.0294). Notably, RMW is the only factor with a significant coefficient (−0.3299, t = −2.213), implying that firms with lower profitability tend to contribute more to skewness premiums.

**Table 4:** Regression results of CAPM, FF3, Carhart, and FF5 models.

Dependent variable: P11				
	CAPM	FF3	Carhart	FF5
Mkt	0.0355	0.0346	0.0154	−0.0006
(t-value)	(0.4630)	(0.4300)	(0.1970)	(−0.0090)
SMB		−0.0265	−0.0255	−0.1727
(t-value)		(−0.2490)	(−0.2420)	(−1.4020)
HML		0.0310	0.0226	0.0145
(t-value)		(0.2270)	(0.1570)	(0.1030)
MOM			−0.1297*	−0.0808
(t-value)			(−1.8610)	(−1.1410)
CMA				0.0548
(t-value)				(0.3430)
RMW				−0.3299**
(t-value)				(−2.2130)
Intercept (α)	1.0123***	1.0074***	1.0710***	1.0985***
(t-value)	(3.9780)	(3.9970)	(4.1220)	(4.2190)
Observations	240	240	240	240
R2	0.0023	0.0029	0.0138	0.0294

Note: *p < 0.1; **p < 0.05; ***p < 0.01.

Despite including additional factors, none of the models fully explains the return pattern of P11, as evidenced by the consistently high and significant alpha estimates. This suggests that skewness risk represents an independent dimension of risk that existing factor models do not capture. The results align with the prior literature indicating that skewness-based anomalies persist even after accounting for standard risk factors. To test H3, we employ the two-stage Fama-MacBeth (1973) regression approach. This method allows us to examine whether skewness risk is systematically priced in the cross-section of stock returns after controlling for the market risk. In the first stage, we estimate each stock’s market beta using a rolling CAPM regression with a 24-month window. The second stage conducts cross-sectional regressions each month to estimate the coefficients γ_Mkt_ and γ_skew_ based on Equation (9). Finally, the time-series average of the estimated coefficients γ_Mkt_ and γ_skew_ are computed, and their statistical significance is assessed using a t-test.

Table 5 presents the summary statistics for the Fama-MacBeth regression over the full sample period from 2005 to 2024. The results indicate that the average market risk premium γ_Mkt_ is negative (−0.1664) but statistically insignificant (t = −0.7273, p = 0.4678), suggesting that market risk does not have a strong cross-sectional explanatory power in this dataset. In contrast, the coefficient of skewness (γ_skew_) is negative and highly significant (−0.0021, t = −4.0245, p = 0.0001). The statistical significance of γ_skew_ provides robust evidence that stocks with higher skewness tend to have lower expected returns, which is consistent with the lottery preference hypothesis and the argument that investors are willing to accept lower returns in exchange for the possibility of extremely positive outcomes.

**Table 5:** Summary of Fama-MacBeth regression results.

Coefficients	γ_Mkt_	γ_skew_
Mean	−0.1664	−0.0021
Standard Error	0.2289	0.0005
Median	−0.1560	−0.0017
Minimum	−24.5516	−0.0349
Maximum	14.9572	0.0228
t-test	−0.7273	−4.0245
p-value	0.4678	0.0001

These findings support the hypothesis H3, which states that skewness is a priced risk factor in the Thai stock market. The negative and significant γ_skew_ suggests that investors discount stocks with high skewness, leading to lower average returns, and reinforcing the existence of a skewness-related anomaly in asset pricing. The results are consistent with prior studies that highlight the impact of skewness preference in emerging markets and provide further empirical evidence that standard risk factors fail to capture the cross-sectional variation in stock returns.

### 4.4. Discussion

This study provides robust and consistent evidence that skewness is a priced risk factor in the Thai stock market. Our portfolio analysis demonstrates that a simple long-short strategy (buying stocks with the most negative past skewness and shorting those with the most positive skewness) generates an economically and statistically significant excess return of 1.017% per month. This finding supports our first hypothesis (H1) and confirms a systematic negative relationship between skewness and expected returns, consistent with the risk compensation hypothesis.

Importantly, this skewness premium is not absorbed by traditional asset pricing models. As shown in Table 4, the P11 portfolio maintains a significantly positive alpha across all models, including the CAPM, Fama-French three-, Carhart four-, and Fama-French five-factor models. This persistence lends empirical support to our second hypothesis (H2) and indicates that conventional factors—such as market beta, size, value, momentum, profitability, and investment—fail to capture the return variation attributable to skewness. Furthermore, Fama-MacBeth regressions confirm that skewness remains a statistically significant cross-sectional predictor even after controlling for market risk, reinforcing our third hypothesis (H3).

However, this study relies on a moment-based estimator of total skewness, which, while transparent and widely used, conflates two economically distinct components: systematic skewness (coskewness) and idiosyncratic skewness. Systematic skewness reflects an asset’s contribution to the asymmetry of a well-diversified portfolio and has been shown in the three-moment CAPM [12] to command a premium. Conversely, idiosyncratic skewness, which captures firm-specific asymmetry, is often linked to behavioral biases such as lottery preferences, leading investors to overpay for stocks with high positive skewness [8,25]. Because our analysis does not disentangle these channels, the pricing anomaly we document may stem from either—or both—mechanisms. Future research using higher-quality data and advanced estimators should explicitly test these competing theories.

Another key limitation is the assumption of frictionless markets. Our long- and short-term portfolios implicitly assume unrestricted short-selling and negligible transaction costs. However, these assumptions are unrealistic in Thailand. The Thai capital market imposes significant short-selling constraints, and retail investors—who dominate trading volume—often lack access to derivatives or leverage. These frictions may prevent arbitrageurs from fully exploiting the skewness premium, resulting in persistent mispricing. We acknowledge this limitation explicitly in the revised discussion and cite empirical works that demonstrate how market frictions contribute to the persistence of pricing anomalies in emerging markets [23].

These findings challenge the semi-strong form of the Efficient Market Hypothesis, which posits that all publicly available information—including higher-order moments such as skewness—should be rapidly and fully incorporated into asset prices. In theory, rational investors equipped with symmetric access to information would arbitrage such anomalies. However, in practice, the Thai market exhibits structural inefficiencies that limit the adjustment mechanism. The dominance of retail investors, relatively low institutional participation, behavioral biases such as a preference for lottery-like stocks, and regulatory barriers—particularly short-selling restrictions—undermine the ability of prices to accurately reflect asymmetry-related risks. Therefore, our results, align more closely with behavioral finance perspectives and segmented-market hypotheses than with strict EMH adherence. Similar inefficiencies have been documented in other markets in the literature. For instance, Apriyadi [26] found that temporary economic uncertainty significantly impacts Shariah-compliant stock returns in Indonesia. While these stocks are structured to reduce exposure to such uncertainty, their post-shock recovery is slower than that of conventional stocks, suggesting incomplete information transmission and market frictions even in supposedly resilient segments. Likewise, Khoa and Huynh [27] provide evidence that the Vietnamese stock market is informationally inefficient, further reinforcing the argument that emerging markets often deviate from the expectations of classical models of market efficiency.

From a practical perspective, these findings have implications for investors, policy makers, and researchers. The anomaly associated with skewness can be partially attributed to behavioral biases, particularly investors’ preference for lottery-like stocks—which offer a small chance of an extremely high payoff despite having poor average performance. According to Boyer, Mitton [8], Brunnermeier, Gollier [25], such stocks with high positive idiosyncratic skewness tend to be overpriced because investors overweigh small-probability extreme gains. This mispricing is especially persistent in emerging markets such as Thailand, where retail investors dominate and speculative tendencies are more pronounced. Consequently, the negative skewness premium observed in this study arises because investors irrationally bid up the prices of highly skewed “lottery” stocks, leading to lower future returns, while neglecting negatively skewed stocks that ultimately earn higher compensation for downside risk. For investors, incorporating skewness signals—especially by avoiding stocks with excessive positive skewness—could improve risk-adjusted performance. For policymakers, understanding the behavioral underpinnings of return asymmetry may help design market reforms that mitigate speculative distortions, promote investor education, and reduce frictions that prevent informed arbitrage. These insights also support further research into the role of behavioral anomalies in shaping asset prices under institutional constraints and the heterogeneous investor’s behavior.

We conducted several checks to test the robustness of our results. First, we replaced the conventional skewness estimator with the Doane and Seward [28] bias-corrected measure and found that the skewness premium persisted. Second, we regressed the long-short portfolio’s returns on liquidity and downside beta [29], and neither factor explained the alpha. These results indicate that the skewness premium is distinct from other risk dimensions.

The limitations of this study point to a clear agenda for future research. First, future studies should decompose skewness into systematic and idiosyncratic components using institutional-grade datasets. Second, as Thailand’s derivatives market matures, option-implied skewness—offering forward-looking insight—should be used to validate the findings. Third, researchers could explore interaction effects between skewness and other anomalies, such as size, beta, or sentiment. Finally, incorporating transaction costs and short-sale constraints directly into strategy simulations will help assess whether the skewness anomaly is arbitrageable or simply unexploitable due to real-world trading frictions.

## 5. Conclusion

This study investigates the role of skewness risk in asset pricing, focusing on its impact on stock returns in the Thai stock market from 2005 to 2024. By constructing long-short portfolios based on skewness exposure, we analyze whether skewness is priced in the cross-section of stock returns. The findings reveal that stocks with low skewness earn significantly higher returns than those with high skewness, supporting the risk compensation hypothesis. The statistically significant positive return of the long-short portfolio (P11) suggests that investors demand compensation for bearing negative skewness risk. Furthermore, the multifactor regression analysis shows that traditional asset pricing models, including CAPM, FF3, FF4, and FF5, fail to fully explain the skewness premium. The persistence of significant alpha across all models provides strong evidence of an asset pricing anomaly. The results of the Fama-MacBeth regressions further confirm that skewness is a systematically priced risk factor, reinforcing the argument that investors adjust their expected returns based on skewness exposure.

The findings of this study contribute to the growing literature on asset pricing anomalies and provide empirical support for integrating skewness as a risk factor. In an emerging market context, where inefficiencies and behavioral biases are more pronounced, skewness pricing effects appear stronger, highlighting the importance of considering higher-order moments in asset pricing frameworks. This study has several implications for investors, portfolio managers, and policymakers. For investors, incorporating skewness-based strategies can enhance portfolio performance by exploiting mispricing associated with return asymmetry. For policymakers, understanding the role of skewness in asset pricing can inform market regulations and risk management policies that aim to improve market efficiencies.

Future research could extend this analysis by exploring the impact of trading frictions on skewness-based strategies, examining the interaction between skewness and other risk factors, and expanding the study to other emerging markets to assess the generalizability of these findings. Additionally, incorporating alternative measures of skewness, such as option-implied skewness, may provide further insights into the pricing mechanisms of skewness risk.
